# First Insights on the Administration of Insect Oil (Black Soldier Fly Larvae) in the Diet of Juvenile *Onychostoma macrolepis*

**DOI:** 10.3390/ani13030518

**Published:** 2023-02-01

**Authors:** Nina Gou, Kaifeng Wang, Tiezhi Jin, Bin Yang

**Affiliations:** Shaanxi Institute of Zoology, Xi’an 710032, China

**Keywords:** antioxidant status, black soldier fly larvae oil, growth performance, lipid metabolism, *Onychostoma macrolepis*

## Abstract

**Simple Summary:**

Black soldier fly oil has become the focus of attention as an alternative component of fish oil in feed. The purpose of this study was to investigate the effects of different levels of black soldier fly oil replacing fish oil in diets on the growth, health and lipid metabolism of *Onychostoma macrolepis*. The results show that it is feasible to replace fish oil with 25–50% BSFLO in the diet. Supplementing with BSFLO in the diet could improve the antioxidant capacity and reduce fat deposits in *Onychostoma macrolepis*.

**Abstract:**

The use of insect products in aquatic feed is the focus of the aquaculture industry. Black soldier fly larvae oil (BSFLO) has been examined as a potential lipid source for diets for some fish species, but its utilization on *Onychostoma macrolepis* has not been explored. The influences of substituting fish oil (FO) with BSFLO in the diet on growth, biological indicators, approximate composition, serum biochemistry, antioxidant capacity and expression of lipid metabolism genes in juvenile *O. macrolepis* were investigated in an eight-week feeding experiment. Four experimental diets were prepared by replacing 0 (control), 25% (BSFLO-25), 50% (BSFLO-50) and 100% (BSFLO-100) FO with BSFLO, and then randomly assigned to twelve aquariums with ten fish (1.75 ± 0.05 g) in each aquarium. The findings showed that the growth indicators, body composition and serum biochemistry in the BSFLO-25 and BSFLO-50 groups had no statistical differences from those of the control group. The activities of superoxide dismutase (SOD) (91.22–94.96 U/mgprot) and the contents of malondialdehyde (MDA) (1.12–1.16 nmol/mgprot) in the liver appeared to be significantly the highest and the lowest in the BSFLO-25, BSFLO-50 and BSFLO-100 groups (*p* < 0.05). The adipocyte size and intraperitoneal fat index value of fish fed on the BSFLO-100 diet were significantly decreased compared to the control group (*p* < 0.05). The expression levels of lipid catabolism genes pparα, (peroxisome proliferators-activated receptor alpha) and cpt1a (carnitine palmitoyltransferase 1 isoform a) in the BSFLO-100 group were significantly higher than those of the control group (*p* < 0.05). The above results indicated that it was feasible to replace 25–50% dietary FO with BSFLO in juvenile *O. macrolepis*. Dietary BSFLO supplementation could enhance the antioxidant capacity of the liver and suppress intraperitoneal fat accumulation in *O. macrolepis*. The use of other insect oils in the diets of this species will be evaluated in future research.

## 1. Introduction

Aquaculture production is on the rise due to the growing global consumer demand for aquatic products. The expansion of intensive farming has contributed to the rising demand for aquatic feed raw materials, particularly protein and lipid sources. More and more limited marine resources (fish meal, fish oil) cannot meet the requirements of aquatic feed production. Therefore, the selection of feed raw material substitutes is a critical demand of the sustainable development of the aquaculture industry. To reduce the impact on marine ecology and lower material costs, fish oil (FO) is often replaced by vegetable oil in commercial feed manufacturing. It is well known that terrestrial animal oils such as lard and tallow are widely used in the feed industry. Alternative oil components derived from insects could be another way to address this problem, at least partially replacing FO in aquatic feed. Insect sources are rich in protein and fat, their fatty acid composition can be controlled by selecting suitable culturing substrates and they do not contain any anti-nutrient factors [1]. In addition, insect-rearing has a more gentle environmental footprint due to more efficient land use, less greenhouse gases and low-carbon production methods [2].

Numerous insect species have been used in aquaculture to prepare feed components, among which the black soldier fly (*Hermetia illucens*) is considered as one of the most promising insect resources [3,4]. Black soldier flies have the advantages of fast growth and reproduction, and high feed efficiency. This species also has the ability to bio-recycle nutriments from cheap agricultural and sideline products and organic garbage [5]. Black soldier flies’ culturing can be a sustainable way to produce feed ingredients. A previous study reported that both black soldier fly larvae meal (BSFLM) and black soldier fly larvae oil (BSFLO) could provide good nutriments for fish [6]. The protein content of BSFLM can reach 40%, and it also contains several different amino acids such as lysine and leucine [7]. In various fish feeding trials, BSFLM has succeeded in replacing fish meal to some extent [8,9,10]. The supplementation of 20% BSFLM in the diet of Ide (*Leuciscus idus*) had good effects on fish culturing and health [11]. Recent research showed that the supplement of 30–60% BSFLM in fish meal-free diets was beneficial to the growth and welfare of rainbow trout (*Oncorhynchus mykiss*) [12]. Gougbedji et al. reported that replacing fish meal with high levels of BSFLM could be considered without fear of negative effects on fish [13]. BSFLO is rich in fat (24–45%) and also contains a variety of fatty acids such as lauric acid and linoleic acid [2,14]. The supplementation of saturated fatty acids (SFAs) in fish feeds, especially short-chain and medium-chain SFAs, could accelerate their utilization as a fuel source without aggravating tissue fat deposition due to their rapid digestion and metabolism [15,16]. Therefore, SFAs are preferentially employed for β oxidation to increase the selective reserve of polyunsaturated fatty acids (PUFAs), which are then retained for the rest of the physiological activities [17]. 

To date, the application of BSFLO in feed has been reported in some fish species. There are some different findings regarding the potential of BSFLO as an alternative to dietary oil sources at different gradient levels. In an earlier study, Li et al. [14] reported that gradually increasing dietary BSFLO supplemental levels (0, 25, 50, 75 and 100%) had no adverse impacts on the growth and nutrient retention of Jian carp (*Cyprinus carpio* var. Jian). Furthermore, dietary BSFLO could lower the fat deposition of Jian carp by regulating the expression of lipid metabolism genes. In mirror carp (*Cyprinus carpio* var. specularis), BSFLO was found to be superior to other insect oil sources (yellow mealworm oil or silkworm pupae oil), reflecting in its positive effects on growth, lipid catabolism and immune activity [16]. In addition, the dietary supplementation of 50–100% BSFLO rich in n-3 long-chain PUFA had beneficial effects on the growth and health of mirror carp [18]. Good growth performance and high feed efficiency were discovered in rainbow trout when the diet contained 10% BSFLO [6]. Similarly, substituting FO or soybean oil with 100% BSFLO exhibited no effects on rainbow trout, including the growth, approximate composition, nutrient deposition and expression of fatty acid metabolism genes [19]. A recent study by Mexican researchers Maldonado-Othón et al. showed that BSFLO could effectively substitute 30% of FO in the diet of *Totoaba macdonaldi* [20]. These findings indicated that it was possible to substitute FO with BSFLO in the diet, but the acceptance degree of BSFLO varied greatly among different fish species, which might be attributed to the species–specific difference. 

*Onychostoma macrolepis* is a newly farmed freshwater omnivorous fish, and its rearing industry is becoming increasingly popular in China. Compared with other freshwater fish, there are few reports on the nutritional requirements of *O. macrolepis*, especially in terms of dietary oil source substitution. Previous research reported that linseed oil or mixed vegetable oil (*Schizochytrium* sp. oil: soybean oil: linseed oil = 1:1:1) could be used to replace FO as a dietary lipid source for *O. macrolepis* without negative effects [21]. Until now, there have been no studies on the dietary administration of BSFLO in *O. macrolepis.* This study provides the first insights on the use of BSFLO in the diet of juvenile *O. macrolepis*. The aim of this research was to comprehensively estimate the effect of replacing FO with BSFLO in the diet of juvenile *O. macrolepis* through a series of indicators, including growth and biological indexes, approximate composition, serum biochemical indices, antioxidant activity and lipid metabolism gene expression.

## 2. Materials and Methods

### 2.1. Ethical Statement

This rearing experiment was conducted in compliance with the guidelines for the care and use of laboratory animals in China and approved by the Ethics Committee of Shaanxi Institute of Zoology (Approval No. L22D004A51).

### 2.2. Experimental Diets

Four semi-purified diets were prepared to satisfy the requirement of crude protein (37%) and crude fat (9%) for *O. macrolepis* (Table 1). With a 100% FO-containing diet as the control (FO), the other three diets were gradually supplemented with 25, 50 and 100% BSFLO (Huafei, Bio-Deve Co., Shenzhen, China) to replace FO (LYSI, Co., Reykjavik, Iceland) (designated BSFLO-25, BSFLO-50 and BSFLO-100, respectively). The dietary ingredients were mixed with water and extruded into cold pellet with a diameter of 2 mm. These feed pellets were dried at room temperature for at least 24 h before storing them in a −20 °C refrigerator. The fatty acid composition of all diets is shown in Table 2.

### 2.3. Fish and Feeding Conditions

Juvenile *O. macrolepis* were purchased from a fishing farm near Tai’an, China, and delivered to the institute in foam boxes. The fish were fed commercial pellets (37% crude protein, 10% crude fat) three times a day until vision satiety. After two weeks of acclimation, one hundred and twenty *O. macrolepis* juveniles (1.75 ± 0.05 g) were randomly selected and distributed in twelve recirculating glass aquariums (50 cm × 30 cm × 45 cm, length × width × height, ten fish/aquarium). The experiment was randomly designed with three replicates per treatment and lasted for eight weeks. Four treatments of fish were hand-fed three times a day (8:30, 12:30 and 16:30) to obvious satiation with each of the randomized trail feeds. Fish feces and unconsumed feed were removed by siphoning daily. The temperature (22 ± 2 °C), dissolved oxygen (6.3 ± 0.2 mg/L) and pH (7.0 ± 0.1) of water were all within the acceptable ranges of this species during the feeding trial. A 12 h light:12 h dark photoperiod was kept throughout the experiment.

### 2.4. Sampling

At the end of the growth experiment, the fish were fasted for 24 h before sampling. The experimental fish (three fish/aquarium, nine fish/treatment) were anesthetized with MS-222 (60 mg/L), and then body mass and overall length were recorded. Blood was obtained from the caudal vein (nine fish/treatment) by syringes and then clotted at 4 °C for 6 h. The blood specimens were then centrifuged at 3000 rpm for 10 min, and serum was collected. Serum samples were frozen with liquid nitrogen and placed in a −80 °C refrigerator for biochemical detection. After blood collecting, these fish (nine fish/treatment) were dissected immediately, and then the liver, muscle and intraperitoneal fat tissue from the fish were removed and weighed, respectively. The liver and muscle samples (nine fish/treatment) were frozen with liquid nitrogen and kept at a −80 °C refrigerator for important composition detection (three fish/treatment), immune activity (three fish/treatment) and fluorescent quantitation (three fish/treatment) analysis. The remaining samples were kept as spares. Whole fish (six fish/treatment) were randomly taken and placed in a −20 °C refrigerator for approximate composition detection. 

### 2.5. Methods of Detection and Analysis

#### 2.5.1. Approximate Composition Detection

The approximate composition of the diets and tissues was determined in accordance with the official analytical method of AOAC [22]. Moisture contents were measured using an oven at 105 °C for 6 h. The contents of ash were obtained by burning in a Muffle furnace at 550 °C for 4 h. The contents of crude protein and crude fat were detected based on the Kjeldahl method and Soxhlet extraction, respectively. 

#### 2.5.2. Determination of Fatty Acid Composition in Diet

The method of Folch et al. [23] was used to extract the lipids of diets. Fatty acid methyl ester (FAME) was prepared according to previous research [22]. In brief, 1 mL hexane was added to dissolve the lipids, and then 1 mL methanol KOH (0.4 M) was added for 1 h methyl esterification. Subsequently, 2 mL double distilled water was added to divide the mixture into two layers. Then, the upper layer was taken for a further test. Tridecanoic acid was selected as a marker. Gas chromatography (7890A, Agilent Technologies, Palo Alto, CA, USA) was performed to obtain FAMEs using a CD-2560 capillary column (100 m length, 0.25 mm inner diameter and 0.2 μm thickness). 

#### 2.5.3. Serum Biochemical Analysis

Serum samples were analyzed for levels of total protein (TP, g/L), albumin (ALB, g/L), globulin (GLO, g/L), cholesterol (CHOL, mmol/L), triglyceride (TG, mmol/L), high-density lipoprotein (HDL, mmol/L), low-density lipoprotein (LDL, mmol/L), aspartate aminotransferase (AST, U/L) and alanine aminotransferase (ALT, U/L) on an automatic biochemical analyzer (TBA-120FR, Toshiba Co., Ltd., Tokyo, Japan) using Shanghai Kehua biochemical diagnosis kits (Shanghai Kehua Bio-Engineering Co., Ltd., Shanghai, China).

#### 2.5.4. Antioxidant Activity Assays in the Liver

The activities of superoxide dismutase (SOD), glutathione peroxidase (GPx) and catalase (CAT) in the liver were determined using Nanjing Jiancheng kits (Nanjing Jiancheng Bioengineering Institute, Nanjing, China) following the instructions of the kits. The content of malondialdehyde (MDA) in the liver was measured by a special assay kit (Nanjing Jiancheng Bioengineering Institute, Nanjing, China). All the above test procedures were operated strictly according to the manufacturer’s instructions.

#### 2.5.5. Intraperitoneal Fat Histology

Histological samples were collected during the dissection (two fish/aquarium). The intraperitoneal fat sample was rinsed with normal saline and fixed with a formalin solution buffered by 4% phosphate. After 24 h of washing with running water, the fixed samples were transferred to ethanol for dehydration, infiltrated with xylene and then embedded with paraffin, based on the histological standard technique. Sections were serially cut at a thickness of 5 μm by a rotating microtome (Leica, Wetzlar, Germany), and then stained using hematoxylin and eosin. Histological specimens were observed using a microscope (Olympus, Tokyo, Japan). The average fat cell size of each image was quantified in Photoshop following the method of Osman et al. [24]. The mean value of five non-overlapping images was computed for each treatment.

#### 2.5.6. Gene Expression

Liver samples were used to assess the expression of lipid metabolism genes. Primer Express 5.0 was used to design the primers, and the gene sequences are shown in Table 3. The total RNA was extracted from the liver using RNAiso Plus (TaKaRa, Dalian, China). The quantity of RNA was detected by a NanoDrop 1000 spectrophotometer (Thermo Scientific, Waltham, MA, USA). The purity of RNA was assessed by testing the absorption ratio of OD260/OD280. The total RNA was reverse-transcribed to the cDNA using the PrimeScript™RT reagent kit (TaKaRa, Dalian, China). Real-time quantitative PCR (RT-qPCR) analyses were performed by a CFX Connect RT-qPCR instrument (Bio-Rad, Hercules, CA, USA) using abm^®^EvaGreen qPCR MasterMix- no dye kit (ABM, Richmond, Canada). All amplification reactions were conducted in triplicate. The volume of the reaction system was 20 μL, including 10.0 μL abm^®^EvaGreen qPCR MasterMix-ROX (2×), 1 μL cDNA (100 ng/μL), 0.8 μL forward primer (0.4 μM), 0.8 μL reverse primer (0.4 μM) and 7.4 μL sterilized distilled water. The thermal cycle condition was 95 °C, 10 min; 94 °C, 15 s, 40–45 cycles; 60 °C, 1 min. By the end of each amplification, melting curves were analyzed to determine the specificity of the PCR products. The β-actin was used to normalize the detected genes, and the 2^−ΔΔCt^ method was selected to calculate gene expression levels [25].

### 2.6. Statistical Analysis

SPSS 22.0 software (IBM, Armonk, NY, USA) was used for the statistical analysis. All data were analyzed by a one-way analysis of variance (ANOVA) after prior tests of normality and homogeneity of variances. A Duncan post hoc test was selected to examine whether there were significant differences among the treatment groups, and *p* < 0.05 was considered as the significant difference level. The experimental values were presented as mean and SEM.

## 3. Results

### 3.1. Growth and Biometric Indices

The growth performance and biometric indices of juvenile *O. macrolepis* are presented in Table 4. No mortality was observed among the dietary groups throughout the study. At the end of the rearing trial, the replacement of FO with BSFLO by 25% and 50% elicited no statistically significant effect on the final weight (FW), weight gain (WG) and specific growth rate (SGR) of juvenile *O. macrolepis* compared with the control group (*p* > 0.05). In comparison with the fish in the control group, the fish in the BSFLO-100 group displayed significantly lower growth variables and feed intake (FI) (*p* < 0.05). All treatment groups showed a similar feed conversion ratio (FCR), and there was no significant difference among them. Compared with the control group, the intraperitoneal fat index (IPFI) of the fish was significantly decreased in the BSFLO-100 group (*p* < 0.05). No significant differences were observed in the viscera index (VI) and hepatosomatic index (HSI) of juvenile *O. macrolepis*.

### 3.2. Approximate Composition of Whole Fish, Muscle and Liver 

The approximate composition of whole fish, muscle and liver is shown in Table 5. At the end of the growth trial, the crude fat content of the whole body in the BSFLO-100 group was significantly reduced compared to the control group (*p* < 0.05). No significant differences were discovered in the composition of the muscle and liver of juvenile *O. macrolepis*.

### 3.3. Serum Biochemical Indices 

All serum biochemical indexes among treatments are presented in Table 6. The significantly lower serum TG concentration of fish was apparent in the BSFLO-100 group compared to the control group (*p* < 0.05). The highest levels of ALT and AST observed in the BSFLO-100 group, and both of the two indicators, were significantly different from those in the control group (*p* < 0.05). No significant differences were found in other serum indexes such as ALB, TP, GLO, CHOL, HDL and LDL among all treatment groups.

### 3.4. Antioxidant Activities 

The hepatic antioxidant indexes of juvenile *O.macrolepis* are shown in Table 7. Compared with the fish in the control group, the fish in the BSFLO-25, BSFLO-50 and BSFLO-100 groups exhibited significantly higher hepatic SOD activities (*p* < 0.05). In the liver, the contents of MDA in the BSFLO-25, BSFLO-50 and BSFLO-100 groups were significantly decreased compared to the control group (*p* < 0.05). No significant differences were observed in the activities of GPx and CAT between the treatment groups.

### 3.5. Intraperitoneal Fat Morphology

**Figure 1:**
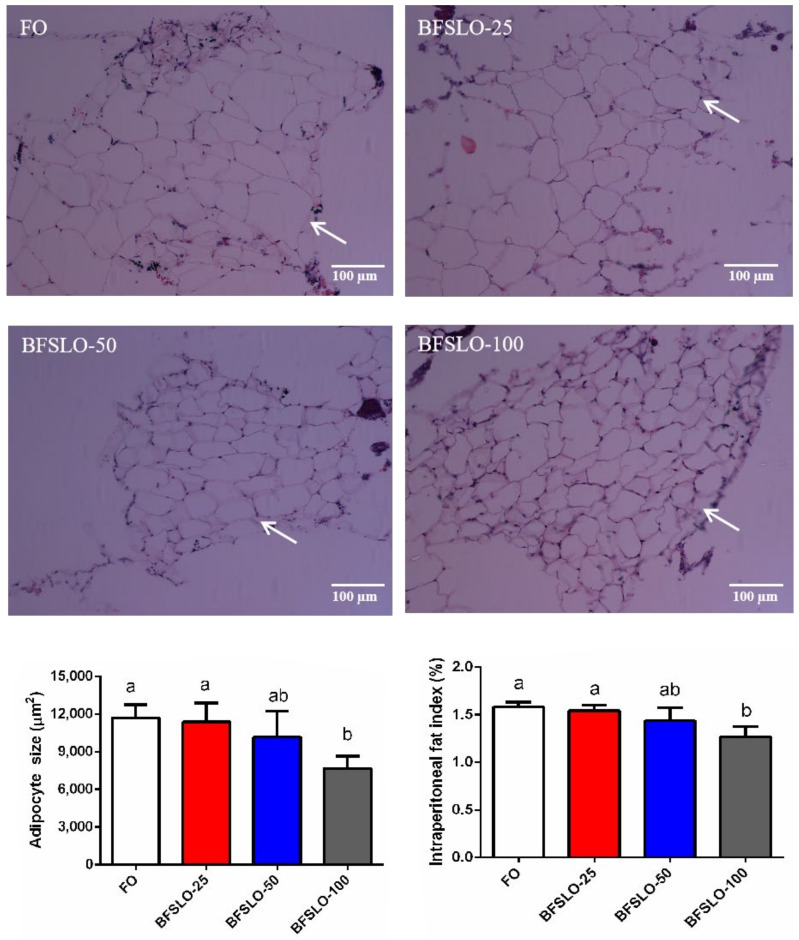
Morphology, the size of adipocyte and intraperitoneal fat index (IPFI) in *O. macrolepis* fed the four experimental diets with different gradient levels of BSFLO (25%, 50% and 100%) replacing FO (control) for eight weeks. Adipocyte size and IPFI value of fish in the BFSLO-100 group were significantly lower than those in the control group (*p* < 0.05). Data are expressed as mean and SEM. Values with different superscripts (a, b, ab) in the same column are significantly different (one-way ANOVO) (*p* < 0.05) The mean value of five non-overlapping images was computed for each treatment.

To determine the effect of dietary BSFLO supplementation on fat deposition in *O. macrolepis*, we measured the adipocyte size and IPFI values (Figure 1). At the end of the trial, the adipocyte size and IPFI value of the fish in the BSFLO-100 group were significantly lower than those in the control group (*p* < 0.05). There were no significant differences found in adipocyte development and IPFI among the BSFLO-25, BSFLO-50 and control groups.

### 3.6. mRNA Expression of Lipid Metabolism-Related Genes 

**Figure 2:**
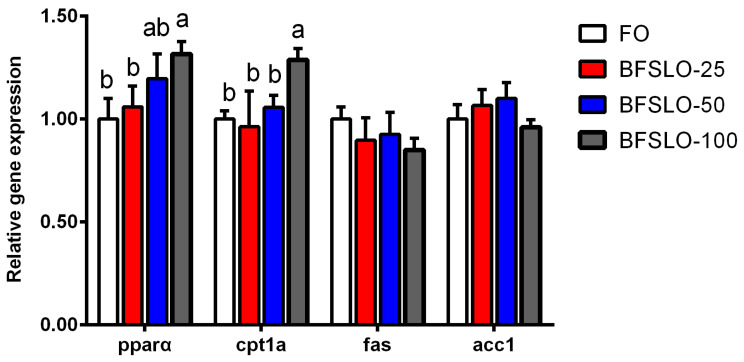
The relative expression of genes involved in lipid metabolism of intraperitoneal fat in *O. macrolepis* fed the four experimental diets with different gradient levels of BSFLO (25%, 50% and 100%) replacing FO (control) for eight weeks. Data are expressed as mean and SEM. Values with different superscripts (a, b, ab) in the same column are significantly different (one-way ANOVO) (*p* < 0.05). pparα, peroxisome proliferators-activated receptor alpha. cpt1a, carnitine palmitoyltransferase 1 isoform a. fas, fatty acid synthase. acc1, acetyl-CoA carboxylase 1.

The mRNA expression of peroxisome proliferators-activated receptor alpha (pparα), carnitine palmitoyltransferase 1 isoform a (cpt1a), fatty acid synthase (fas) and acetyl-CoA carboxylase 1 (acc1) in intraperitoneal fat of *O. macrolepis* are presented in Figure 2. There were no significant differences in the mRNA expression levels of pparα and cpt1a between the BSFLO-25, BSFLO-50 and control groups. With the dietary BSFLO supplementation levels elevated, the pparα expression levels of *O. macrolepis* were presented to tend to increase, and exhibited significantly higher levels in the BSFLO-100 group (*p* < 0.05). The mRNA expression of cpt1a in the BSFLO-100 group was significantly higher compared with the control group (*p* < 0.05). No statistically significant differences appeared in the transcriptional levels of fas and acc1 between the treatment groups.

## 4. Discussion

Insects are an important part of the natural bait resources in *O. macrolepis*, including Coleoptera, Trichoptera and Diptera. As far as we know, there has been no research utilizing BSFLO as a dietary lipid source for *O. macrolepis*. In this study, the results demonstrated that 25–50% of BSFLO could be supplemented in the diet without affecting the survival and growth of juvenile *O. macrolepis*. Similar results were found in *T. macdonaldi* [20] and rainbow trout [6], suggesting that BSFLO could partially substitute for FO at appropriate supplemental levels. When 100% BSFLO was used to replace FO, poor growth performance presented in *O. macrolepis*, characterized by lower WG and SGR. The slow growth of fish could be caused by various factors. One reason might be the feed ingestion of the fish. A significant reduction in feed intake was observed in the BSFLO-100 group, indicating that high inclusion contents of BSFLO could result in lower feed acceptability. The results were consistent with a report by Caimi et al. [8], who suggested that the decrease in feed consumption could lead to a decline in fish growth variables. The fatty acid profile of the diet could not meet the essential fatty acid requirement for the growth of juvenile *O. macrolepis* when dietary FO was substituted with 100% BSFLO [26]. This might be another reason. In a review, Tocher [27] mentioned that poor growth could be a consequence of deficient or insufficient essential fatty acid in fish diets. Conversely, previous research recorded that the diet containing up to 75–100% BSFLO had no influence on the FW and SGR of Jian carp [14]. Furthermore, Fawole et al. [19] reported that there were no significant changes in the WG and SGR of rainbow trout when FO was completely replaced by BSFLO in the diet. Differences in fish species selected in the various experiments might account for the inconsistencies reported in the published literature. The crude protein content of *O. macrolepis* was not influenced by dietary BSFLO inclusion levels, indicating that the body protein was endogenous adaptive regulation and relatively stable. The significantly lower crude fat of the fish was recorded in the BSFLO-100 group, inconsistent with a previous study conducted on *T. macdonaldi* in which the body fat was not impacted by the levels of BSFLO substituting FO [20]. The cause for these differences might be due to the various life histories of *O. macrolepis* (freshwater fish) and *T. macdonaldi* (marine species), or changes in ingredients among BSFLO products. 

Excess fat accumulation could affect visceral mass and relative liver size. In the present study, there were no differences in HSI and VSI values among the BSFLO supplemental groups and the control group, suggesting that no abnormal lipid accumulation was observed when BSFLO was supplemented in the diet. This result was in accordance with a previous study, suggesting that the replacement of FO with BSFLO in the diet had no significant effect on the HSI and VSI values of fish [20]. On the other hand, the VSI value of rainbow trout increased significantly when BSFLO was substituted for FO in the diet [19]. The current study exhibited a significantly lower IPFI value in the BSFLO-100 group. The finding was consistent with previous research, suggesting that high supplemental levels of BSFLO had a certain effect on alleviating intraperitoneal fat deposition [14]. The cause for this result might be that BSFLO contained high levels of medium-chain fatty acids, and these components were thought to have an obesity-inhibiting effect in rats [28]. The size of the adipocytes (representing the fat amount per fat cell), together with the number of adipocytes (which is associated with apoptosis), determine the quality of the adipose tissue. The adipocyte size of intraperitoneal fat can be detected by histological observation. In this study, the diminution of adipocyte size in the BSFLO-100 group might lead to the decrease in IIPFI. It is generally recognized that the size of adipocytes correlated with the regulation of lipid metabolism genes. Pparα and cpt1a play important roles in lipid catabolism, while lipid biosynthesis can be regulated by fas and acc1 [29]. In the current study, significantly elevated transcription levels of pparα and cpt1a appeared in the BSFLO-100 groups, indicating that the inhibitory effect of BSFLO on adipocyte development might be mediated by promoting the expression of lipid catabolism genes. Similar findings were reported on Jian carp [14] and mirror carp [16]. In the meantime, the expression of fas and acc1 in intraperitoneal fat of *O. macrolepis* had no significant differences among the treatment groups. 

In various rearing experiments, a blood biochemical analysis was considered to be an important reference for assessing the impacts of dietary component substitution on the health condition of different fish species [30,31,32]. In this research, improving the dietary BSFLO inclusion level to 100% could decrease the serum TG concentration, which might be connected with the existence of medium-chain fatty acids. It was found that medium-chain fatty acids could lower the TG content in rats [28]. On the contrary, replacing soybean oil with BSFLO in the diet had no effect on serum TG concentrations of Jian carp [14]. This could be attributed to differences in the control diet used in these trials, and vegetable oils probably contained plant active ingredients [19]. AST and ALT are usually released into the blood when hepatocytes are threatened, and elevated activities of these two aminotransferases are potentially dangerous to the liver function of fish [30]. In the present study, the activities of AST and ALT in the BSFLO-100 group were significantly higher than those in the control group, indicating that the complete replacement of FO by BSFLO could affect the health of the liver. The cause for the reduced aminotransferase activity in the BSFL-100 group is unclear, and further research is needed to better understand the effect of BSFLO on the immune response of *O. macrolepis*.

Several common antioxidant enzymes, including SOD, GPx and CAT, are beneficial to preserve fish cells from damage induced by reactive oxygen species and play vital roles in the immune activity of fish [33,34]. MDA, as the end product of lipid peroxidation, is regarded as a marker of oxidative stress [35]. In the current study, SOD activities in the liver of fish in the BSFLO groups with different gradient levels (BSFLO-25, BSFLO-50 and BSFLO-100 groups) were significantly higher than those of the control group. This result suggested that the dietary supplementation of BSFLO could improve the antioxidant capacity of fish [19]. Ratti et al. reported that the diet containing 20% black soldier fly prepupae increased the expression level of immune-related genes in the gut of rainbow trout [36]. The diet supplemented with 30% BSFLM had no adverse effect on the liver and intestine antioxidant status of *Argyrosomus regius* [37]. The replacement of 50% fish meal with BSFLM resulted in a slight increase in antioxidant activity, characterized by a decrease in MDA, but did not alter the fish health [38]. In this study, significantly lower MDA contents of *O. macrolepis* were observed in the three groups supplemented with BSFLO, indicating that the BSFLO-based diets contained lower PUFA levels and tended less toward peroxidation compared to the control diet (FO as a fat source) [39].

## 5. Conclusions

In summary, the results of this experiment indicated that it was feasible to substitute 25–50% FO with BSFLO without affecting the growth, biological indicators, approximate composition and serum biochemical indices of juvenile *O. macrolepis*. Supplementation of BSFLO in the diet could not only improve the antioxidant capacity of the liver, but also suppress the adipocyte development in *O. macrolepis*. The next step will be to assess other insect oils and appropriate supplemental levels in the diets of *O. macrolepis* for practical purposes.

## Figures and Tables

**Table 1:** Ingredients (g/kg) and proximate composition of the four experimental diets with different gradient levels of BSFLO (25%, 50% and 100%) replacing FO (control).

Ingredients	FO	BSFLO-25	BSFLO-50	BSFLO-100
Casein	350	350	350	350
Gelatin	104	104	104	104
Dextrin	270	270	270	270
Microcrystalline cellulose	115	115	115	115
Vitamin premix ^1^	10	10	10	10
Mineral misture ^2^	40	40	40	40
Carboxymethylcellulose	20	20	20	20
BHT	1	1	1	1
FO ^3^	90	67.5	45	0
BSFLO ^4^	0	22.5	45	90
Total	1000	1000	1000	1000
Dry matter (%)	90.09	89.52	90.03	89.71
Crude protein (%)	37.11	37.25	36.97	37.34
Crude fat (%)	9.35	9.19	9.22	9.16
Ash (%)	10.82	10.13	10.79	10.99
NFE ^5^ (%)	32.81	32.95	33.05	32.22
GE ^6^ (MJ/kg)	18.37	18.36	18.32	18.24

^1^ Vitamin premix (mg/kg diet): vitamin C, 200; thiamine, 10; riboflavin, 20; vitamin A, 3000 IU; vitamin E, 50 IU; vitamin D3, 1500 IU; menadione, 10; pyridoxine HCl, 10; cyanocobalamin, 0.02; biotin, 1.0; calcium pantothenate, 40; folic acid, 5; niacin, 20; inositol, 400; choline chloride, 2000; and cellulose was used as a carrier. ^2^ Mineral mixture (g/100 g total mineral): KAl(SO_4_) 0.159, CaCO_3_ 18.101; Ca(H_2_PO_4_)_2_ 44.601, CoCl 0.070, MgSO_4_ 5.216, MnSO_4_·H_2_O 0.070, KCl 16.553, KI 0.014, ZnCO_3_ 0.192, NaH_2_PO_4_ 13.605, Na_2_SeO_3_ 0.006, CuSO_4_.5H_2_O 0.075, Ferric citrate 1.338. ^3^ FO, fish oil. ^4^ BSFLO, black soldier fly larvae oil. ^5^ NFE(%), nitrogen-free extract = 100 − (crude protein% + crude fat% + ash% + moisture%). ^6^ GE (MJ/kg), gross energy = [23.9 × crude protein (g/kg) + 39.8 × crude fat (g/kg) + 17.6 × NFE (g/kg)]/1000.

**Table 2:** Fatty acid composition (mg/g) of the four experimental diets with different gradient levels of BSFLO (25%, 50% and 100%) replacing FO (control).

Fatty Acids	FO	BFSLO-25	BFSLO-50	BFSLO-100
C12:0	0.00	6.30	12.46	25.25
C14:0	1.94	1.77	1.79	3.14
C16:0	17.65	18.80	17.63	19.61
C18:0	5.78	5.21	4.15	4.01
C16:1n-7	1.10	1.31	1.26	1.97
C18:1n-9	13.98	13.86	14.19	14.05
C18:2n-6	4.14	6.01	8.43	10.15
C18:3n-6	0.68	0.70	0.82	1.07
C20:3n-6	0.50	0.20	0.70	1.00
C20:4n-6	0.73	1.10	1.17	1.26
C18:3n-3	2.94	2.69	1.53	0.27
C20:3n-3	0.28	0.24	0.18	0.00
C20:5n-3	21.27	17.07	12.93	0.00
C22:6n-3	14.53	12.05	10.96	0.00
∑SFA ^1^	25.37	32.08	36.03	52.01
∑MUFA ^2^	15.08	15.17	15.45	16.02
∑n-6 PUFA	6.05	8.01	11.12	13.48
∑n-3 PUFA	39.02	32.05	25.60	0.27
∑PUFA ^3^	45.07	40.06	36.72	13.75
n-3/n-6 PUFA	6.45	4.00	2.30	0.02

^1^ SFA, saturated fatty acid. ^2^ MUFA, monounsaturated fatty acid. ^3^ PUFA, polyunsaturated fatty acid.

**Table 3:** Forward and reverse primers’ sequences used for real-time quantitative PCR.

Gene	Gene Bank Accession No.	Sequence
pparα ^1^	MG735214.1	F:TGACATGGAGGTGCTGGAGGAC
		R:TGCTGCTGTGCTGTTGCTCTG
cpt1a ^2^	MH553647	F:CTCAGACGGTGTTCAGTGCCATC
		R:TCCAGCCGTGATAGGACAAGAGG
fas ^3^	MG735211.1	F:ATCCACAGAGCCACCATCCTACC
		R:CAAGTCCAGCATCCTCCAAGACAC
acc1 ^4^	MG735212.1	F:AGGTGGTACGGATGGCTGCTC
		R:GACGGTGCTGGACGCTGTTG
β-actin^5^	JN254630.1	F:TGACCCACACTGTACCCATC
		R:CGGACAATTTCACTCTCGGC

^1^ pparα, peroxisome proliferators-activated receptor alpha. ^2^ cpt1a, carnitine palmitoyltransferase 1 isoform a. ^3^ fas, fatty acid synthase. ^4^ acc1, acetyl-CoA carboxylase 1. ^5^ β-actin, house keeping gene.

**Table 4:** Growth, biological indicators and feed utilization efficiency of *O. macrolepis* fed the four experimental diets with different gradient levels of BSFLO (25%, 50% and 100%) replacing FO (control) for eight weeks.

	FO	BFSLO-25	BFSLO-50	BFSLO-100
IBW ^1^ (g)	1.75 ± 0.04	1.74 ± 0.02	1.76 ± 0.03	1.77 ± 0.02
FBW ^2^ (g)	9.26 ^a^ ± 0.20	9.07 ^a^ ± 0.18	8.81 ^ab^ ± 0.17	8.39 ^b^ ± 0.14
WG ^3^ (%)	429.57 ^a^ ± 14.27	422.59 ^a^ ± 15.93	401.34 ^ab^ ± 4.08	373.78 ^b^ ± 3.30
SGR ^4^ (%/day)	2.98 ^a^ ± 0.05	2.95 ^a^ ± 0.06	2.88 ^ab^ ± 0.02	2.78 ^b^ ± 0.02
FI ^5^ (g/fish)	11.61 ^a^ ± 0.15	11.28 ^ab^ ± 0.12	11.01 ^ab^ ± 0.26	10.68 ^b^ ± 0.21
FCR ^6^	1.55 ± 0.06	1.54 ± 0.03	1.56 ± 0.01	1.61 ± 0.02
VSI ^7^ (%)	8.86 ± 0.26	8.66 ± 0.31	8.55 ± 0.21	8.19 ± 0.13
HSI ^8^ (%)	1.51 ± 0.03	1.54 ± 0.03	1.55 ± 0.05	1.51 ± 0.08
IPFI ^9^ (%)	1.58 ^a^ ± 0.03	1.54 ^a^ ± 0.03	1.44 ^ab^ ± 0.13	1.27 ^b^ ± 0.11

Data are expressed as mean and SEM. Values with different superscripts (a, b, ab) in the same row are significantly different (one-way ANOVO) (*p* < 0.05). ^1^ IBW, initial body weight. ^2^ FBW, final body weight. ^3^ WG, weight gain (%) = (final body weight − initial body weight) × 100/initial body weight. ^4^ SGR, specific growth rate (%/day) = (Ln final weight − Ln initial weight) × 100/days. ^5^ FI, feed intake (g/fish) = total dry feed given (g)/number of fish. ^6^ FCR, feed conversion ratio = feed consumed (g)/weight gain (g). ^7^ VSI, viscera index (%) = viscera weigh × 100/body weight. ^8^ HSI, hepatopancreas index (%) = hepatopancreas weigh × 100/body weight. ^9^ IPFI, intraperitoneal fat index (%) = intraperitoneal fat weight × 100/body weight.

**Table 5:** Proximate composition (% wet weight) in whole body, muscle and liver of *O. macrolepis* fed the four experimental diets with different gradient levels of BSFLO (25%, 50% and 100%) replacing FO (control) for eight weeks.

	FO	BFSLO-25	BFSLO-50	BFSLO-100
Whole dody				
Moisture	70.27 ± 0.88	70.29 ± 0.76	70.33 ± 0.96	71.2 ± 0.67
Crude protein	16.44 ± 0.67	16.25 ± 0.69	16.37 ± 0.84	16.13 ± 0.30
Crude lipid	10.82 ^a^ ± 0.19	10.41 ^a^ ± 0.16	10.36 ^a^ ± 0.15	9.63 ^b^ ± 0.13
Ash	2.31 ± 0.14	2.55 ± 0.12	2.49 ± 0.15	2.85 ± 0.22
Muscle				
Moisture	77.18 ± 0.61	77.55 ± 0.68	77.2 ± 0.53	77.94 ± 0.85
Crude protein	19.15 ± 0.29	19.26 ± 0.26	19.45 ± 0.21	19.36 ± 0.23
Crude lipid	1.41 ± 0.14	1.38 ± 0.08	1.25 ± 0.06	1.18 ± 0.05
Ash	1.33 ± 0.08	1.29 ± 0.08	1.27 ± 0.10	1.24 ± 0.08
Liver				
Moisture	61.06 ± 1.03	61.33 ± 0.84	61.45 ± 1.12	62.64 ± 0.69
Crude protein	15.74 ± 0.65	15.53 ± 1.00	15.2 ± 0.58	15.35 ± 0.54
Crude lipid	21.59 ± 0.81	21.85 ± 1.63	21.81 ± 1.80	20.11 ± 1.68
Ash	1.08 ± 0.02	1.13 ± 0.08	1.06 ± 0.03	1.05 ± 0.03

Data are expressed as mean and SEM. Values with different superscripts (a, b, ab) in the same row are significantly different (one-way ANOVO) (*p* < 0.05).

**Table 6:** Serum biochemical indices of *O.macrolepis* fed the four experimental diets with different gradient levels of BSFLO (25%, 50% and 100%) replacing FO (control) for eight weeks.

	FO	BFSLO-25	BFSLO-50	BFSLO-100
ALB ^1^ (g/L)	6.14 ± 0.10	6.13 ± 0.14	6.15 ± 0.21	6.21 ± 0.10
GLO ^2^ (g/L)	11.76 ± 0.51	12.04 ± 0.73	10.95 ± 0.41	11.25 ± 0.21
TP ^3^ (g/L)	17.9 ± 0.55	18.17 ± 0.59	17.1 ± 0.61	17.47 ± 0.28
CHOL ^4^ (mmol/L)	1.65 ± 0.07	1.75 ± 0.08	1.65 ± 0.08	1.64 ± 0.10
TG ^5^ (mmol/L)	1.73 ^a^ ± 0.10	1.74 ^a^ ± 0.14	1.58 ^ab^ ± 0.03	1.36 ^b^ ± 0.03
HDL ^6^ (mmol/L)	0.56 ± 0.05	0.64 ± 0.06	0.63 ± 0.06	0.62 ± 0.06
LDL ^7^ (mmol/L)	0.43 ± 0.05	0.41 ± 0.03	0.39 ± 0.02	0.36 ± 0.03
AST ^8^ (U/L)	169.87 ^b^ ± 4.29	166.67 ^b^ ± 3.84	168.27 ^b^ ± 6.58	188.67 ^a^ ± 4.91
ALT ^9^ (U/L)	83.00 ^b^ ± 4.59	81.50 ^b^ ± 3.14	82.10 ^b^ ± 3.86	96.07 ^a^ ± 2.81

Data are expressed as mean and SEM. Values with different superscripts (a, b, ab) in the same row are significantly different (one-way ANOVO) (*p* < 0.05). ^1^ ALB, albumin. ^2^ GLO, globulin. ^3^ TP, total protein. ^4^ CHOL, cholesterol. ^5^ TG, triglyceride. ^6^ HDL, high-density lipoprotein. ^7^ LDL, low-density lipoprotein. ^8^ AST, aspartate aminotransferase. ^9^ ALT, alanine aminotransferase.

**Table 7:** Antioxidant indexes in the liver of *O.macrolepis* fed the four experimental diets with different gradient levels of BSFLO (25%, 50% and 100%) replacing FO (control) for eight weeks.

	FO	BFSLO-25	BFSLO-50	BFSLO-100
SOD ^1^ (U/mgprot)	81.18 ^b^ ± 2.33	94.96 ^a^ ± 2.77	94.34 ^a^ ± 3.27	91.22 ^a^ ± 1.29
GPx ^2^ (U/mgprot)	20.91 ± 1.46	28.98 ± 4.82	33.15 ± 4.22	26.15 ± 3.77
CAT ^3^ (U/mgprot)	17.6 ± 0.92	19.3 ± 0.73	18.08 ± 1.62	19.74 ± 2.15
MDA ^4^ (nmol/mgprot)	1.48 ^a^ ± 0.11	1.15 ^b^ ± 0.08	1.16 ^b^ ± 0.06	1.12 ^b^ ± 0.06

Data are expressed as mean and SEM. Values with different superscripts (a, b, ab) in the same row are significantly different (one-way ANOVO) (*p* < 0.05). ^1^ SOD, superoxide dismutase. ^2^ GPx, glutathione peroxidase. ^3^ CAT, catalase. ^4^ MDA, malondialdehyde.

## Data Availability

All data are included in the article.

## References

[1] Spranghers T., Ottoboni M., Klootwiik C., Ovyn A., Deboosfere S., De Meulenaer B., Michiels J., Eeckhout M., De Clerq P., De Smet S. (2017). Nutritional composition of black soldier fly (*Hermetia illucens*) prepupae reared on different organic waste substrate. J. Sci. Food Agric..

[2] Benzertiha A., Kierończyk B., Rawski M., Mikolajczak Z., Urbański A., Nogowski L., Józefiak D. (2020). Insect fat in animal nutrition—A review. Ann. Anim. Sci..

[3] Tran G., Heuzé V., Makkar H.P.S. (2015). Insects in fish diets. Anim. Front..

[4] Mohan K., Rajan D.K., Muralisankar T., Ganesan A.R., Sathishkumar P., Revathi N. (2022). Use of black soldier fly (*Hermetia illucens* L.) larvae meal in aquafeeds for a sustainable aquaculture industry: A review of past and future needs. Aquaculture.

[5] Gougbedji A., Agbohessou P., Lalèyè P., Francis F., Megido R. (2021). Technical basis for the small-scale production of black soldier fly, *Hermetia illucens* (L. 1758), meal as fish feed in Benin. J. Agric. Food Res..

[6] Dumas A., Raggi T., Barkhouse J., Lewis E., Weltzien E. (2018). The oil fraction and partially defatted meal of black soldier fly larvae (*Hermetia illucens*) affect differently growth performance, feed efficiency, nutrient deposition, blood glucose and lipid digestibility of rainbow trout (*Oncorhynchus mykiss*). Aquaculture.

[7] Xu Y., Zhang J., Song Z., Sun Y. (2014). Optimization of extraction of proteins from larvae of the black soldier fly, *Hermetia illucens* (Diptera: Stratiomyidae), using response surface methodology. Acta. Entomol. Sin..

[8] Caimi C., Renna M., Lussiana C., Bonaldo A., Gariglio M., Meneguz M., Dabbou S., Schiavone A., Gai F., Elia A.C. (2020). First insights on Black Soldier Fly (*Hermetia illucens* L.) larvae meal dietary administration in Siberian sturgeon (*Acipenser baerii* Brandt) juveniles. Aquaculture.

[9] Kishawy A., Mohammed H., Zaglool A., Attia M., Hassan F., Roushdy E., Ismail T., Ibrahim D. (2022). Partial defatted black solider larvae meal as a promising strategy to replace fish meal protein in diet for Nile tilapia (*Oreochromis niloticus*): Performance, expression of protein and fat transporters, and cytokines related genes and economic efficiency. Aquaculture.

[10] Hender A., Siddik M.A.B., Howieson J., Fotedar R. (2021). Black soldier fly, *Hermetia illucens* as an alternative to fishmeal protein and fish oil: Impact on growth, immune response, mucosal barrier status, and flesh quality of juvenile barramundi, *Lates calcarifer* (Bloch, 1790). Biology.

[11] Homska N., Kowalska J., Bogucka J., Ziółkowska E., Rawski M., Kierończyk B., Mazurkiewicz J. (2022). Dietary Fish Meal Replacement with Hermetia illucens and Tenebrio molitor Larval Meals Improves the Growth Performance and Nutriphysiological Status of Ide (*Leuciscus idus*) Juveniles. Animals.

[12] Cardinaletti G., Marco P.D., Daniso E., Messina M., Donadelli V., Finoia M.G., Petochi T., Fava F., Faccenda F., Contò M. (2022). Growth and Welfare of Rainbow Trout (*Oncorhynchus mykiss*) in Response to Graded Levels of Insect and Poultry By-Product Meals in Fishmeal-Free Diets. Animals.

[13] Gougbedji A., Detilleux J., Lalèyè P.A., Francis F., Megido R.C. (2022). Can Insect Meal Replace Fishmeal? A Meta-Analysis of the Effects of Black Soldier Fly on Fish Growth Performances and Nutritional Values. Animals.

[14] Li S., Ji H., Zhang B., Tian J., Zhou J., Yu H. (2016). Influence of black soldier fly (*Hermetia illucens*) larvae oil on growth performance, body composition, tissue fatty acid composition and lipid deposition in juvenile Jian carp (*Cyprinus carpio* var. Jian). Aquaculture.

[15] Schönfeld P., Wojtczak L. (2016). Short- and medium-chain fatty acids in energy metabolism: The cellular perspective. J. Lipid Res..

[16] Xu X., Ji H., Belghit I., Sun J. (2020). Black soldier fly larvae as a better lipid source than yellow mealworm or silkworm oils for juvenile mirror carp (*Cyprinus carpio* var. specularis). Aquaculture.

[17] Marques V.H., Moreira R.G., Branco G.S., Honji R.M., Rombenso A.N., Viana M.T., Araújo B.C. (2021). Different saturated and monounsaturated fatty acids levels in fish oil-free diets to cobia (*Rachycentron canadum*) juveniles: Effects in growth performance and lipid metabolism. Aquaculture.

[18] Xu X., Ji H., Belghit I., Liland N., Wu W., Li X. (2021). Effects of black soldier fly oil rich in n-3 HUFA on growth performance, metabolism and health response of juvenile mirror carp (*Cyprinus carpio* var. specularis). Aquaculture.

[19] Fawole F., Labh S., Hossain M., Overturf K., Small B., Welker T., Hardy R., Kumar V. (2021). Insect (black soldier fly larvae) oil as a potential substitute for fifish or soy oil in the fish meal-based diet of juvenile rainbow trout (*Oncorhynchus mykiss*). Anim. Nutr..

[20] Maldonado-Othón C., Re-Vega E., Perez-Velazquez M., González-Félix M. (2022). Replacement of fish oil by camelina and black soldier fly larvae oils in diets for juvenile *Totoaba macdonaldi* and their effect on growth, fatty acid profile, and gene expression of pancreatic lipases. Aquaculture.

[21] Gou N.N., Ji H., Zhong M.Z., Chang Z.G., Deng W. (2020). Effects of dietary fish oil replacements with three vegetable oils on growth, fatty acid composition, antioxidant capacity, serum parameters and expression of lipid metabolism related genes in juvenile Onychostoma macrolepis. Aquac. Nutr..

[22] AOAC (2012). Official Methods for Analysis.

[23] Folch J., Lees M., Sloane Stanley G.H. (1957). A simple method for the isolation and purification of total lipids from animal tissues. J. Biol. Chem..

[24] Osman O.S., Selway J.L., Kępczyńska M.A., Stocker C.J., O’Dowd J.F., Cawthorne M.A., Arch J.R., Jassim S., Langlands K. (2013). A novel automated image analysis method for accurate adipocyte quantification. Adipocyte.

[25] Livak K.J., Schmittgen T.D. (2001). Analysis of relative gene expression data using realtime quantitative PCR and the 2^−ΔΔCt^ method. Methods.

[26] Gou N.N., Ji H., Chang Z.G., Zhon M.Z., Deng W. (2020). Effects of dietary essential fatty acid requirements on growth performance, fatty acid composition, biochemical parameters, antioxidant response and lipid related genes expression in juvenile *Onychostoma macrolepis*. Aquaculture.

[27] Tocher D.R. (2010). Fatty acid requirements in ontogeny of marine and freshwater fish. Aquac. Res..

[28] Han J., Hamilton J.A., Kirkland J.L., Corkey B.E., Guo W. (2003). Medium chain oil reduces fat mass and down regulates expression of adipogenic genes in rats. Obes. Res..

[29] Zheng J.L., Luo Z., Zhu Q.L., Tan X.Y., Chen Q.L., Sun L.D., Hu W. (2013). Molecular cloning and expression pattern of 11 genes involved in lipid metabolism in yellow catfish *Pelteobagrus fulvidraco*. Gene.

[30] Fawole F.J., Adeoye A.A., Tiamiyu L.O., Ajala K.I., Obadara S.O., Ganiyu I.O. (2020). Substituting fishmeal with *Hermetia illucens* in the diets of African catfish (*Clarias gariepinus*): Effects on growth, nutrient utilization, haematophysiological response, and oxidative stress biomarker. Aquaculture.

[31] Sankiana Z., Khosravia S., Kimb Y.O., Lee S.M. (2018). Effects of dietary inclusion of yellow mealworm (*Tenebrio molitor*) meal on growth performance, feed utilization, body composition, plasma biochemical indices, selected immune parameters and antioxidant enzyme activities of mandarin fish (*Siniperca scherzeri*) juveniles. Aquaculture.

[32] Abdel-Tawwab M., Khalil R.H., Metwally A.A., Shakweer M.S., Khallaf M.A., Abdel-Latif H.M.R. (2020). Effects of black soldier fly (*Hermetia illucens* L.) larvae meal on growth performance, organs-somatic indices, body composition, and hematobiochemical variables of European sea bass, Dicentrarchus labrax. Aquaculture.

[33] Moutinho S., Pedrosa R., Magalhães R., Oliva-Teles A., Parisi G., Peres H. (2021). Black soldier fly (*Hermetia illucens*) pre-pupae larvae meal in diets for European seabass (*Dicentrarchus labrax*) juveniles: Effects on liver oxidative status and fillet quality traits during shelf-life. Aquaculture.

[34] Yadav A.K., Rossi W., Habte-Tsion H.M., Kumar V. (2020). Impacts of dietary eicosapentaenoic acid (EPA) and docosahexaenoic acid (DHA) level and ratio on the growth, fatty acids composition and hepaticantioxidant status of largemouth bass (*Micropterus salmoides*). Aquaculture.

[35] Koruk M., Taysi S., Savas M.C., Yilmaz O., Akcay F., Karakok M. (2004). Oxidative stress and enzymatic antioxidant status in patients with nonalcoholic steatohepatitis. Ann. Clin. Lab. Sci..

[36] Ratti S., Zarantoniello M., Chemello G., Giammarino M., Palermo F.A., Cocci P., Mosconi G., Tignani M.V., Pascon G., Cardinaletti G. (2023). Spirulina-enriched Substrate to Rear Black Soldier Fly (*Hermetia illucens*) Prepupae as Alternative Aquafeed Ingredient for Rainbow Trout (*Oncorhynchus mykiss*) Diets: Possible Effects on Zootechnical Performances, Gut and Liver Health Status, and Fillet Quality. Animals.

[37] Guerreiro I., Castro C., Serra C.R., Coutinho F., Couto A., Peres H., Pousão-Ferreira P., Gasco L., Gai F., Oliva-Teles A. (2022). Oxidative Stress Response of Meagre to Dietary Black Soldier Fly Meal. Animals.

[38] Melenchón F., Mercado E., Pula H., Cardenete G., Barroso F.G., Fabrikov D., Lourenço H.M., Pessoa M.F., Lagos L., Weththasinghe P. (2022). Fishmeal Dietary Replacement Up to 50%: A Comparative Study of Two Insect Meals for Rainbow Trout (*Oncorhynchus mykiss*). Animals.

[39] Elia A., Capucchio M., Caldaroni B., Magara G., Dorr A., Biasato I., Biasibetti E., Righetti M., Pastorino P., Prearo M. (2018). Influence of *Hermetia illucens* meal dietary inclusion on the histological traits, gutmucin composition and the oxidative stress biomarkers in rainbow trout (*Oncorhynchus mykiss*). Aquaculture.

